# Increased half-life and enhanced potency of Fc-modified human PCSK9 monoclonal antibodies in primates

**DOI:** 10.1371/journal.pone.0183326

**Published:** 2017-08-17

**Authors:** Yijun Shen, Hua Li, Li Zhao, Gang Li, Ben Chen, Qingsong Guo, Bei Gao, Jinsong Wu, Tong Yang, Li Jin, Yong Su

**Affiliations:** 1 Ministry of Education Key Laboratory of Contemporary Anthropology, Fudan University, Shanghai, China; 2 R&D Department of Genetic Engineering, Shanghai Fudan-Zhangjiang Bio-Pharmaceutical Co., Ltd., Shanghai, China; University of Padova, ITALY

## Abstract

Blocking proprotein convertase subtilisin kexin type 9 (PCSK9) binding to low-density lipoprotein receptor (LDLR) can profoundly lower plasma LDL levels. Two anti-PCKS9 monoclonal antibodies (mAbs), alirocumab and evolocumab, were approved by the FDA in 2015. The recommended dose is 75 mg to 150 mg every two weeks for alirocumab and 140mg every two weeks or 420 mg once a month for evolocumab. This study attempted to improve the pharmacokinetic properties of F0016A, an IgG1 anti-PCKS9 mAb, to generate biologically superior molecules. We engineered several variants with two or three amino acid substitutions in the Fc fragment based on prior knowledge. The Fc-modified mAbs exhibited increased binding to FcRn, resulting in prolonged serum half-life and enhanced efficacy *in vivo*. These results demonstrate that Fc-modified anti-PCKS9 antibodies may enable less frequent or lower dosing of antibodies by improved recycling into the blood.

## Introduction

Proprotein convertase subtilisin/kexin type 9 (PCSK9), also known as neural apoptosis-regulated convertase 1 (NARC1), is a serine protease of the secretory subtilase family that has profound effects on plasma low-density lipoprotein-cholesterol (LDL-C) levels through its ability to mediate LDL receptor (LDLR) degradation [1–3]. PCSK9 binds to the epidermal growth factor-like repeat A (EGF-A) domain of LDLR on the surface of hepatocytes. The PCSK9-LDLR complexes are then internalized to lysosomes, thereby preventing LDLR from recycling to the cell surface [4–6]. LDLR is the primary receptor that clears circulating LDL, and therefore the decrease in LDLR levels mediated by PCSK9 results in higher blood levels of LDL-C. The link between PCSK9 and plasma LDL-C levels was first established by the discovery of missense mutations in PCSK9 in patients with an autosomal dominant form of familial hypercholesterolemia [7]. These mutations, such as D374Y, were speculated to result in a gain-of-function of PCSK9 that reduces LDLR levels in the liver, resulting in high plasma levels of LDL-C. Subsequently, the Atherosclerosis Risk In Community (ARIC) study and other population studies showed that PCSK9 loss-of-function mutations resulted in lower plasma LDL-C levels and a dramatically reduced risk of coronary heart disease (CHD) [8–10]. The loss of PCSK9 appears to have no adverse consequences [11]. Thus, the PCSK9 protein is regarded as a new therapeutic target to lower plasma levels of LDL, and pharmacological PCSK9 inhibition for lipid lowering might be safe.

A number of monoclonal antibodies (mAbs) against PCSK9 are currently in clinical trials for the treatment of dyslipidemia and atherosclerotic cardiovascular disease. By inhibiting the binding of PCSK9 to LDLR, anti-PCSK9 mAbs increase the number of LDLRs available to clear LDL, thereby lowering LDL-C levels [12]. A meta-analysis of 24 clinical trials demonstrated that PCSK9 mAbs can reduce cholesterol, cardiac events and all-cause mortality [13]. Alirocumab and evolocumab were the first 2 drugs to be approved by the Food and Drug Administration, in 2015. A phase 2 randomized controlled trial demonstrated that alirocumab reduces LDL-C in a dose-dependent manner when given every 4 weeks (the percentage changes in LDL-C were -28.9, -31.54, and -42.53 for doses of 150, 200, and 300 mg given every 4 weeks, respectively) in patients with heterozygous familial hypercholesterolemia, but a sawtooth pattern suggesting LDL rebound was observed. Compared with 4-week dosing, dose concentrations of 150 mg every 2 weeks appear optimal (67.90% reduction) and continue to show benefit [14]. Obviously, dosing frequency is important in terms of the LDL-C response. Similarly, there is a dose-response relationship with total cholesterol, non-high-density lipoprotein cholesterol (HDL-C), and ApoB. Again, the every-2-week regimen is more effective at lowering total cholesterol, non-HDL-C, and ApoB [14]. These dose and duration effects are similar to those observed with evolocumab [15]. Alirocumab and evolocumab yield striking LDL-C reductions over 10 to 78 weeks (26%-67%) [16, 17]. However, as with many novel specialty drugs, the promise of better results with PCSK9 inhibitors comes with a high price tag [18]. Therapeutic antibodies with an extended half-life might prove valuable in antibody therapy and offer the potential benefit of lower frequency of administration and treatment costs.

The serum half-life of IgGs is regulated by the neonatal Fc receptor (FcRn). FcRn is a heterodimer comprising a transmembrane α chain and a soluble β2-microglubulin and mainly mediates both transcytosis of maternal IgG to the fetus or neonate and IgG homeostasis in adults [19]. The binding of IgG to FcRn is pH-dependent; IgG binds to FcRn under an acidic environment (pH 6.0) and is released under physiological pH [20]. By binding to FcRn in endosomes, IgGs are salvaged from lysosomal degradation and recycled to the circulation. Several studies have demonstrated a correlation between the binding affinity of IgGs to FcRn and their serum half-lives [21]. Improved affinity for FcRn is known to extend antibody half-life *in vivo*. Pharmacokinetics study in rhesus monkeys showed that the half-lives of T250Q/M428L variant OST577-IgG_2_M3 (-27 days) and OST577-IgG1 (-35 days), which have increased binding affinity to human and monkey FcRn at pH 6.0 but no change in binding affinity at pH 7.5, were approximately 1.9-fold and 2.5-fold longer than those of the wild-type antibodies (-14.6 days and -14 days, respectively) [22, 23]. The triple mutation M252Y/S254T/T256E (YTE) introduced into the Fc portion of MEDI-524, a humanized anti-respiratory syncytial virus (RSV) mAb, resulted in a 10-fold increase in its binding to both cynomolgus monkey and human FcRn at pH 6.0. MEDI-524-YTE was efficiently released from FcRn at pH 7.4 in both cases and consistently exhibited a nearly 4-fold increase in serum half-life in cynomolgus monkeys compared with MEDI-524 [24]. An engineered version of bevacizumab with the M428L/N434S Xtend double mutant provided an 11-fold improvement in FcRn affinity at pH 6.0 and extended the half-life from 9.7 to 31.1 days, a 3.2-fold improvement in serum half-life relative to native IgG1 in cynomolgus monkeys. Simple allometric scaling extrapolations suggest that this improvement could potentially translate into human half-lives exceeding 50 days [25]. These results show that a modest increase in pH 6 FcRn affinity can result in improved pharmacokinetics in primates.

In this study, several Fc variants of alirocumab, a human IgG1 antibody, were generated with different degrees of improvement in binding to human FcRn at pH 6.0 and pH-dependent binding. The pharmacokinetics (PK) and pharmacodynamics (PD) were evaluated in cynomolgus monkeys.

## Materials and methods

### Expression and purification of human PCSK9

In order to verify whether Fc mutations affect the antigen-binding activity *in vitro*, two human PCSK9 proteins, wild type and its gain-of-function mutant D374Y were expressed and purified. First, Human PCSK9 cDNA was generated by RT-PCR from Hep G2 cells (ATCC, HB-8065), a human liver cancer cell line, and a His_6_ tag was added to the N terminus of the protein. The gene was cloned into the mammalian expression vector pCEP4 (Invitrogen), and the gain-of-function mutation D374Y was then constructed by site-directed mutagenesis using the Fast Mutagenesis System (TransGen Biotech, Inc.) and confirmed by DNA sequencing. The PCSK9 WT and D374Y variant plasmids were transfected separately into human embryonic kidney cells 293F (Gibco) using FreeStyle^™^ MAX Reagent (Invitrogen). Cells that stably expressed the PCSK9 proteins were selected by hygromycin B (Generay). Proteins were purified from culture supernatants using a Ni Sepharose excel column (GE Healthcare).

### Construction, expression, and purification of human PCSK9 mAb variants

The DNA encoding alirocumab, a human IgG1 anti-PCKS9 mAb named F0016A, was generated by gene synthesis (GENEWIZ). The light and heavy chain DNAs were separately subcloned into pEE12.4 expression vectors (Genomeditech). This vector contains the hCMV-MIE promotor to drive expression of the target gene and a GS-deficient marker for stable selection in mammalian cells. The three Fc variants of T251Q/M429L, M429L/N435S and M253Y/S255T/T257E, named F0016B, F0016C and F0016D respectively, were obtained by site-directed mutagenesis of F0016A as described above. According to their labels, both alirocumab (Praluent^®^) and evolocumab (Repatha^®^) were produced in Chinese hamster ovary (CHO) cells. In this study, CHO-K1 (ATCC, CCL-61) stably expressing anti-PCSK9 WT or variant IgGs were created by co-transfection of the light and heavy chains using Lipofectamine 2000 transfection reagent (Invitrogen), followed by selection in HyCell CHO medium (Hyclone) containing 50μM MSX (Sigma). Culture supernatants were collected and purified by rProtein A chromatography (GE Healthcare). For *in vivo* studies, the rProtein A eluate was further purified by Q anion-exchange chromatography (GE Healthcare) and SP cation-exchange chromatography (GE Healthcare). The eluted antibodies were then concentrated. The samples were sterile-filtered, and quality analysis was performed.

### PCSK9 binding studies using surface plasmon resonance

The interactions of the F0016 antibodies with PCSK9 WT and the D374Y variant were monitored by SPR detection using a Biacore T200 instrument (GE Healthcare). Anti-human IgG Fc antibody (Genway) was immobilized onto a Biacore CM5 biosensor chip (GE Healthcare) using an amine coupling kit (GE Healthcare). The running buffer for the binding experiments was HBS-EP+, which included 10 mM HEPES, 150 mM NaCl, 3 mM EDTA, and 0.005% (v/v) surfactant P20, pH 7.4. Flow cells 3 and 4 were activated for 7 min with a 1:1 mixture of 50 mM N-hydroxysuccinimide and 200 mM N-ethyl-N’-dimethylaminopropyl carbodiimide at a flow rate of 10μl/min. Anti-human IgG Fc antibody dissolved in 10 mM sodium acetate, pH 5.0, was injected over flow cells 3 and 4. The surfaces were then blocked with a 7-min injection of 1 M ethanolamine, pH 8.5. Antibodies (F0016A, B, C or D) were injected over the anti-human IgG Fc antibody-coated sensor chip of flow cell 4 at 10μl/min. Flow cell 3 was used as a control surface on which antibodies were not captured. Serial dilutions of PCSK9 WT with concentrations ranging from 0.3125 nM to 10 nM and the D374Y variant with concentrations ranging from 0.25 nM to 8 nM were then injected at a flow rate of 30μl/min (association for 3 min, followed by dissociation for 10 min); 10 mM glycine, pH 1.5, was used to regenerate the chip. The data were globally fit using a 1:1 binding model. The equilibrium dissociation constants (binding affinity, K_D_) for each PCSK9-antibody interaction were determined using Biacore T200 Evaluation Software (GE Healthcare, version 3.0).

### FcRn binding studies using surface plasmon resonance

The interaction of F0016 mAbs with human FcRn (Sino Biological, Inc.) was monitored by SPR detection using a Biacore 3000 instrument (GE Healthcare) as described in the previous paragraph. Streptavidin dissolved in 10 mM sodium acetate, pH 4.26, at a concentration of 5μg/mL was immobilized onto a Biacore CM5 biosensor chip in flow cells 1 and 2 using amine coupling chemistry to reach a density of 1624.5 RU and 2198.0 RU, respectively. FcRn was biotinylated using EZ-Link^®^ Sulfo-NHS-LC-Biotin (Thermo Scientific) according to the manufacturer’s recommendations. Biotinylated FcRn generated using an EZ-Link^™^ Sulfo-NHS-LC-Biotin kit (Thermo Scientific) was prepared at 2 μg/mL in HBS-EP buffer, pH 8.0, and injected for 7 min at a flow rate of 10μl/min. The F0016 antibody dilutions from 0 nM to 10 nM in HBS-EP buffer pH 6.0 were injected at a flow rate of 50μl/min for 3 min, followed by dissociation for 3 min. The chip was regenerated with HBS-EP buffer pH 8.0. Kinetic binding constants for the antibody-FcRn interactions were determined using the Biacore 3000 program from each set of equilibrium binding responses fitted to the 1:1 Langmuir binding model.

### Measurement of pH-dependent binding to FcRn by ELISA

The pH-dependent binding of the PCSK9 mAbs to FcRn was determined by ELISA at pH 6.0 and 7.4. Maxisorp 96-well assay plates (Thermo Scientific) were coated with 10 μg/mL streptavidin (Jackson ImmunoRes) in coating buffer (15 mM Na_2_CO_3_, 35 mM NaHCO_3_, pH 9.6) overnight at 4°C, washed with PBST (PBS supplemented with 0.05% (v/v) Tween 20) pH 7.4, and blocked with blocking buffer (PBS supplemented with 3% BSA) for 2 h at room temperature. Biotinylated FcRn was added at 250 ng/mL and incubated for 1 h at 37°C. After washing three times with PBST pH 8.0, serially diluted F0016 mAbs were added to the plates and incubated for 1 h at 37°C. The plates then were washed with PBST at pH 6.0 and 7.4, respectively. Following incubation with a 1/500 dilution of HRP-conjugated goat anti-human IgG (H+L) antibody (Beyotime Biotechnology) for 1 h at 37°C, the plates were washed again with PBST at pH 6.0 and 7.4, respectively, and developed by the addition of TMB substrate for 20 min at 37°C. EC_50_ values were calculated using a four-parameter logistic fit regression model.

### Studies in cynomolgus monkeys

The study was performed at an AAALAC accredited facility. All animal procedures were performed in compliance with the animal welfare policies and guidelines of SIMM, Chinese Academy of Science. The study protocols were reviewed and approved by the Institutional Animal Care and Use Committee. Ten male cynomologus monkeys (Macaca fascicularis) (Guangdong Landau Biotechnology Co. Ltd., Guangzhou, China) aged 4–5 years, weight 4–7 kg were used in the study. Animals were housed in single sex groups and in the pens with a measurement approximately of 2 m × 1.5 m × 2 m. Ten male animals were housed in five cages as two animals per cage. Animals were offered a set weight of diet (Monkey maintenance feed, Beijing keao xieli feed Co., LTD.) twice daily, in the morning and afternoon. The animals were also given fruit, vegetable, or additional supplements as a form of environmental enrichment. Toys (e.g. platforms, treats) were also provided. Animals had free access to tap water and samples were analyzed every 6 months for specified microorganisms and environmental contaminants. Bedding was provided (Suzhou Zhulin Trade Co. Ltd) and the animal room temperature and relative humidity monitored and maintained at 20°C to 26°C and 40% to 70%, respectively. Rooms were illuminated using a 12 h natural and artificial light /12 h dark cycle. The lights were gradually switched on at 7 am and gradually switched off at 7 pm.

These cynomolgus monkeys were randomized by weight and assigned to five study groups (two animals per group). Group 1 received saline injection instead of antibody drug as control animals. Each animal in the other four groups received a single intravenous dose of 5 mg/kg of F0016A antibody or Fc variant (F0016B, C, D). Blood samples (~1 mL) were collected before and after dosing at 12 h, 24 h, and 72 h and at 7, 10, 14, 17, 21, 24, 28, and 31 days. The samples were incubated at room temperature for 1 h and centrifuged at 4°C and 2000 g for 10 min. The serum was stored at a temperature of -65°C or lower until analysis.

### Bioanalysis of PCSK9 mAb concentrations in serum

Monkey serum sample was collected at the indicated time points. The concentrations of free PCSK9 mAbs in serum samples were detected using a validated ELISA. ELISA 96-well plates (Thermo Scientific) were coated with mouse anti-His tag antibody (CWBIO) at a 1:300 dilution in 0.01 M PB pH 7.4 for 18–24 h at 2–8°C. After a blocking step for 60 ± 5 min in blocking buffer, recombinant human PCSK9 with a His_6_ tag was added at 3μg/mL and incubated for 45 ± 5 min at 37°C. Serially diluted standards (0.625–60 ng/mL) as well as diluted cynomolgus monkey serum samples (minimum 1:40 dilution) were added to the plates and incubated for 60 ± 5 min at 37°C. Bound drug was detected by incubation with a 1/20,000 dilution of HRP-conjugated sheep anti-human IgG Ab (Bethyl Lab) for 2 h ± 5 min at 37°C. Assay volumes were 100μl for all steps except for blocking (200μl). The plates were washed three times with PBST after each step and then developed by the addition of TMB substrate for 5 ± 1 min at 37°C. The reaction was stopped with 50 μl of 2 M H_2_SO_4_, and the absorbance at 450 nm was measured within 10 min using a MD Plus384 plate reader (Molecular Devices Corporation). PCSK9 mAb concentrations were calculated using a four or five parameter logistic fit regression model by SoftMax Pro 6.2.1. The PK assay was validated, the limit of detection (LOD) was 0.625 ng/ml, the lower limit of quantitation (LLOQ) was 1.25 ng/ml, and the upper limit of quantitation (ULOQ) was 40 ng/ml. Concentration versus time data was analyzed with non-compartmental method (winnonlin V 6.3, Pharsight Mountain View, CA). T_1/2_, C_max_, T_max_, AUC_all_, AUC_inf_, V_z_, Cl were estimated PK parameters. Elimination half-life parameter was calculated only towards the linear section while other parameters were calculated towards both the linear and nonlinear section. Data sets were selected randomly to determine λ (lambda) and the elimination half-life (HL_Lambda_z or T_1/2_) automatically by Winnonlin. The maximum concentration (C_max_) was the observed concentration. The area under the curve to infinity (AUC_inf_) was determined by log-linear trapezoidal method and the clearance (Cl_obs) was determined by the equation Dose/AUC.

### Lipid analysis of serum samples

Serum samples were analyzed at different time points for lipid measurements (total cholesterol (CHOL), triglyceride (TG), HDL-C, LDL-C and very low density lipoprotein-cholesterol (VLDL-C) using an automatic biochemical analyzer. PD parameters were estimated with a non-compartment model using WinNonlin Enterprise, version 6.3.

## Results

### Design, construction, and in vitro characterization of the anti-PCSK9 Fc variants

To improve the PK properties of alirocumab, three variants of an IgG1 anti-PCKS9 mAb, referred to as F0016A in this study, with half-life improvements of 2.5- to 5-fold were designed using two or three amino acid substitutions in the Fc fragment according to prior knowledge: T251Q/M429L, M429L/N435S and M253Y/S255T/T257E, named F0016B, F0016C and F0016D respectively. The four mAbs were expressed in CHO-K1 cells, and the harvested cell culture fluid was purified by affinity chromatography, followed by Q anion-exchange chromatography and SP cation-exchange chromatography. The concentrations of the purified mAbs were measured, and the purity and endotoxin were assayed.

### Binding of the PCSK9 mAbs to PCSK9

The affinities of the F0016 mAbs to PCSK9 WT and the D374Y variant were determined using surface plasmon resonance (SPR). As expected, the results showed that the binding of the anti-PCSK9 Fc variants to WT PCSK9 was identical to that of WT Fc (Table 1). The affinities of PCSK9 D374Y to the four mAbs also showed similar results, indicating that the Fc mutations had little or no effect on antigen binding. WT PCSK9 exhibited approximately 2-fold higher affinity to the mAbs than the D374Y variant.

**Table 1:** Binding of F0016 mAbs to PCSK9 WT and the D374Y variant as determined by SPR.

Antigen	Antibody	ka (1/Ms)	kd (1/s)	K_D_ (M)
PCSK9 WT	F0016A	1.581E+6	2.949E-4	1.866E-10
	F0016B	1.697E+6	2.997E-4	1.765E-10
	F0016C	1.675E+6	3.098E-4	1.850E-10
	F0016D	1.666E+6	3.072E-4	1.843E-10
PCSK9-D374Y	F0016A	2.308E+6	8.095E-4	3.507E-10
	F0016B	2.250E+6	8.062E-4	3.583E-10
	F0016C	2.354E+6	8.071E-4	3.428E-10
	F0016D	2.296E+6	7.863E-4	3.425E-10

### Binding of the PCSK9 mAbs to FcRn

FcRn plays an important role in regulating the serum half-lives of IgG mAbs. Binding of the WT and Fc mutant PCSK9 mAbs to human FcRn was evaluated by SPR. The K_D_ of PCSK9 and F0016A, B, C, and D was 4.88, 2.11, 1.76, and 1.91 nM, respectively (Table 2). Compared with the WT antibody, F0016A, all three Fc variants exhibited improved binding to FcRn. The affinity of F0016C to FcRn was 2.8-fold higher than that to F0016A and was higher than those of F0016B and F0016D.

**Table 2:** Binding of F0016 mAbs to FcRn as determined by SPR.

Antibody	ka (1/Ms)	kd (1/s)	K_D_ (M)
F0016A	1.27E+6	6.17E-3	4.88E-9
F0016B	1.43E+6	3.02E-3	2.11E-9
F0016C	1.36E+6	2.40E-3	1.76E-9
F0016D	1.05E+6	2.01E-3	1.91E-9

**Fig 1:**
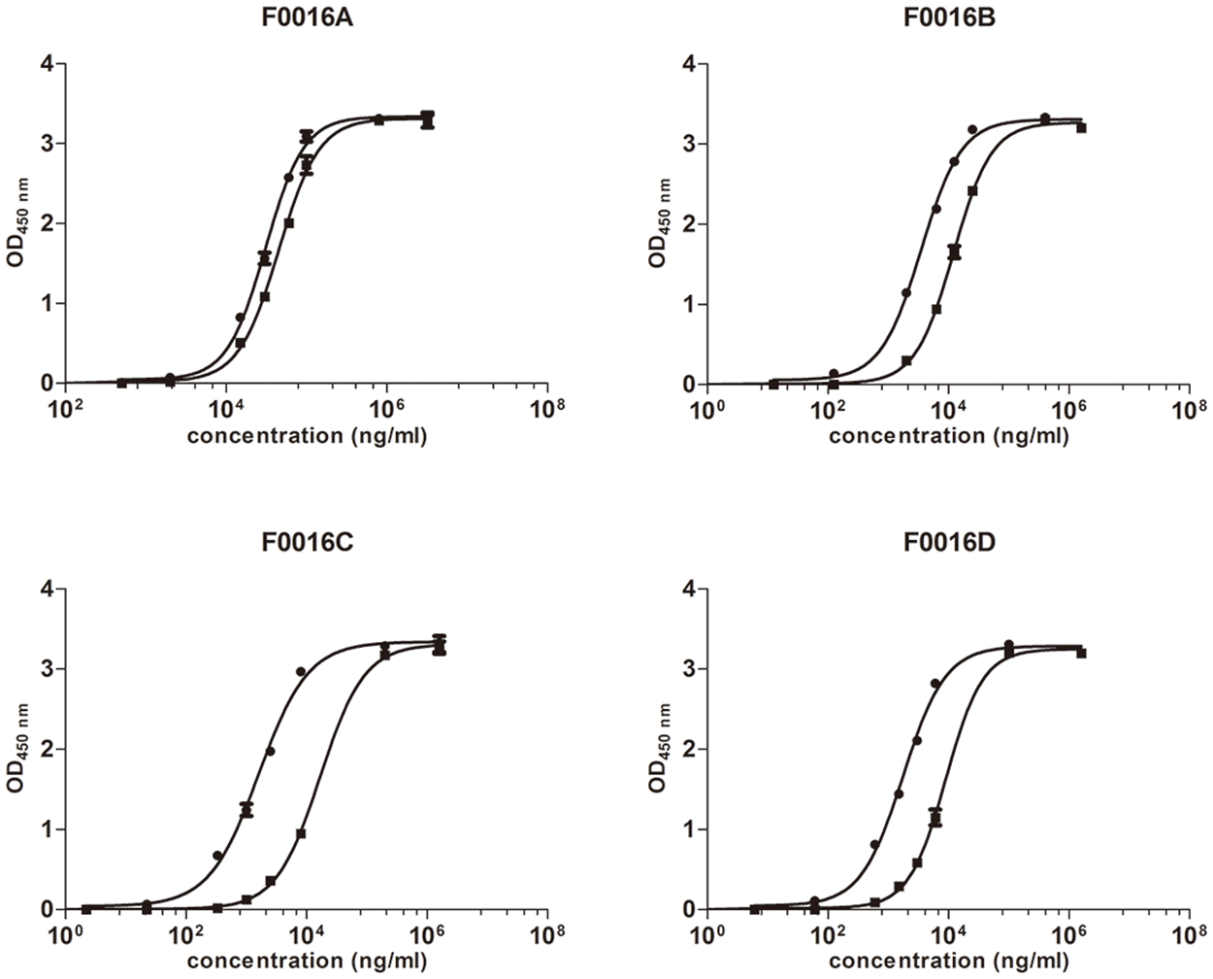
pH-dependent binding of F0016 mAbs to FcRn as assessed by ELISA at pH 6.0 (circles) and pH 7.4 (squares). First, 96-well plates were coated with 10 μg/mL streptavidin overnight at 4°C. Biotinylated FcRn was added at 250 ng/mL and incubated for 1 h at 37°C. Serially diluted F0016 mAbs were added and incubated for 1 h at 37°C, and then the plates were washed with PBST at pH 6.0 or 7.4, respectively. Following incubation with HRP-conjugated goat anti-human IgG (H+L) antibody for 1 h at 37°C, the plates were washed again with PBST at pH 6.0 or 7.4, respectively. The binding curves of F0016 Abs to FcRn were analyzed using a four-parameter logistic fit regression model. Results were shown as mean ± SD (error bars, n = 2).

The binding of IgG to FcRn is markedly pH-dependent; IgG binds to FcRn under mildly acidic conditions and is released under slightly basic conditions. To assess pH dependence, anti-PCSK9 WT and the three Fc variants were evaluated for binding at pH 6.0 and removal by washing at pH 6.0 or 7.4 in an ELISA experiment. All F0016 mAbs exhibited higher binding affinity to FcRn at pH 6.0 than that at pH 7.4 (Table 3, Fig 1). Comparison of the EC_50_ values showed that the binding of F0016A, B, C, and D to FcRn was ~1.46-, 3.45-, 10.43-, and 5.14-fold stronger at pH 6.0 than at pH 7.4, respectively, suggesting that the Fc mutations improved pH dependence and that the pH-dependent binding of F0016C was maximal. The SPR and ELISA both suggested that F0016C might have the longest half-life *in vivo*.

**Table 3:** pH-dependent binding of the F0016 mAbs to FcRn. EC_50_ values were calculated using a four-parameter logistic fit regression model. Results were shown as mean ± SD (n = 2).

Antibody	EC_50_ (μg/ml)	
	pH 6.0	pH 7.4
F0016A	30.45 ± 0.95	45.12 ± 2.11
F0016B	3.47 ± 0.03	12.22 ± 0.55
F0016C	1.56 ± 0.02	16.49 ± 1.43
F0016D	1.75 ± 0.04	8.87 ± 0.59

### The anti-PCSK9 Fc variants show improved PK parameters in non-human primates

**Fig 2:**
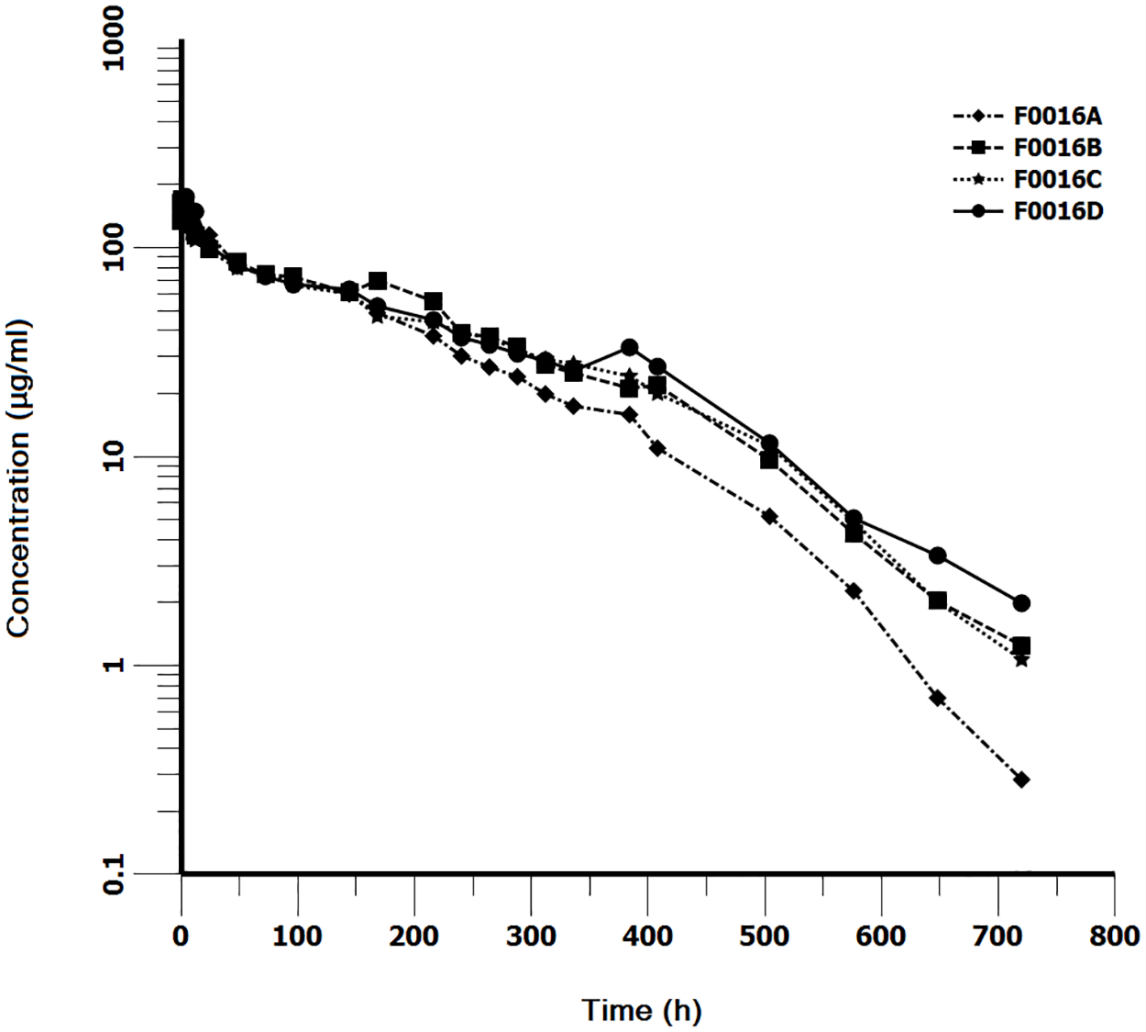
Mean serum concentrations of WT anti-PCSK9 antibody (F0016A) and the Fc variants (F0016B, C, D) versus time after a single intravenous dose of 5 mg/kg to cynomolgus monkeys. First, 96-well plates were coated with mouse anti-His tag antibody at 1:300 dilution for 18–24 h at 2–8°C. Recombinant human PCSK9 with a His_6_ tag was added at 3μg/mL and incubated for 45 ± 5 min at 37°C. Serially diluted standards (0.625–60 ng/mL) as well as diluted cynomolgus monkey serum samples (minimum 1:40 dilution, day 1 ~ day 31) were added and incubated for 60 ± 5 min at 37°C. Bound drug was detected by incubation with HRP-conjugated sheep anti-human IgG Ab for 2 h ± 5 min at 37°C. PCSK9 mAb concentrations were calculated using a four or five parameter logistic fit regression model by SoftMax Pro 6.2.1. The PK assay was validated, the limit of detection (LOD) was 0.625 ng/ml, the lower limit of quantitation (LLOQ) was 1.25ng/ml, and the upper limit of quantitation (ULOQ) was 40 ng/ml. Points, mean of two animals. Points, mean of two animals.

To investigate whether the increased pH-dependent binding to FcRn correlates with longer serum half-lives, the PK profiles of anti-PCSK9 WT and the three Fc variants were examined in cynomolgus monkeys (n = 2 animals per group) following a single intravenous dose of 5 mg/kg. Concentration versus time data was analyzed with non-compartmental method (winnonlin V 6.3, Pharsight Mountain View, CA). T_1/2_, Cmax, Tmax, AUC_all_, AUC_inf_, Vz, Cl were estimated PK parameters. The PK profile of anti-PCSK9 antibodies in the concentration-time curve (Fig 2) showed a characterization mixed non-linear and Michaelis-menten elimination pathways. As the FcRn-mediated elimination is usually non-specific and linear, the terminal elimination slopes may be explained by interaction with target antigen. Therefore, for a purpose to identify the relevance between Fc modification and increased half-lives, elimination half-life parameter was calculated only towards the linear section (0 to 336 h, Fig 2), while other parameters were calculated towards both the linear and nonlinear section (0-720h, Fig 2). Data sets were selected randomly to determine λ (lambda) and the elimination half-life (T_1/2_) automatically by Winnonlin. The maximum concentration (C_max_) was the observed concentration. The area under the curve to infinity (AUC_inf_) was determined by log-linear trapezoidal method and the clearance (Cl_obs) was determined by the equation Dose/AUC.

Calculation values of PK parameters were shown in Table 4. The elimination half-lives (T_1/2_) of F0016 A, B, C and D were 111.39, 125.49, 169.49 and 185.65 h respectively, indicating that the serum half-lives of F0016C and D were longer than the other two. It was difficult to distinct F0016A from F0016B due to large SDs. The apparent volume of distribution (V_Z_) values were similar among the four test groups, suggesting that the administered mAbs were distributed into the circulation in a similar manner. The mean clearance (CL) for F0016A was approximately 0.239 mL/h/kg, which was 1.19-, 1.12- and 1.13-fold faster than those of F0016B, C, and D respectively, indicating a slight decrease in the clearance of Fc variants. The areas under the concentration-time curve (AUCs) for the three mutant F0016 mAbs appeared similar and were approximately 1.1~1.2-fold higher than that of F0016A, indicating a slight increase in the total exposure of Fc variants. Although the small sample size prevented from statistical analysis for this preliminary selection study, the PK parameter values of the four candidates indicated that the increased pH-dependent binding to FcRn were most likely correlated with longer serum half-lives.

**Table 4:** Non-compartment PK parameters of WT anti-PCSK9 antibody (F0016A) and the Fc variants (F0016B, C, D) after a single intravenous dose of 5 mg/kg to cynomolgus monkeys.

Antibody		T_1/2_	C_max_	T_max_	AUC_all_	AUC_inf_	V_ss_	Cl	MRT_last_
		(h)	(μg/mL)	(h)	(h*μg/mL)	(h*μg/mL)	(mL/kg)	(mL/h/kg)	(h)
F0016A	Mean	111.39	178.04	0.375	20954.56	20975.69	35.636	0.239	149.18
	SD	24.20	4.31	0.177	1560.31	1579.38	1.9225	0.018	18.8
F0016B	Mean	125.49	179.7	1.25	24864.46	25007.19	36.183	0.201	177.88
	SD	57.24	18.6	1.06	2274.44	2373.56	2.1425	0.019	25.76
F0016C	Mean	169.49	165.52	0.5	23269.69	23379.19	40.437	0.214	185.39
	SD	9.67	5.02	0	1696.39	1621.94	3.81	0.015	2.37
F0016D	Mean	186.65	192.24	2.125	25053.97	25311.93	38.025	0.212	181.81
	SD	3.25	36.8	2.652	8928.73	9234.45	5.5695	0.077	36.29

### The anti-PCSK9 Fc variants extend cholesterol lowering in non-human primates

**Fig 3:**
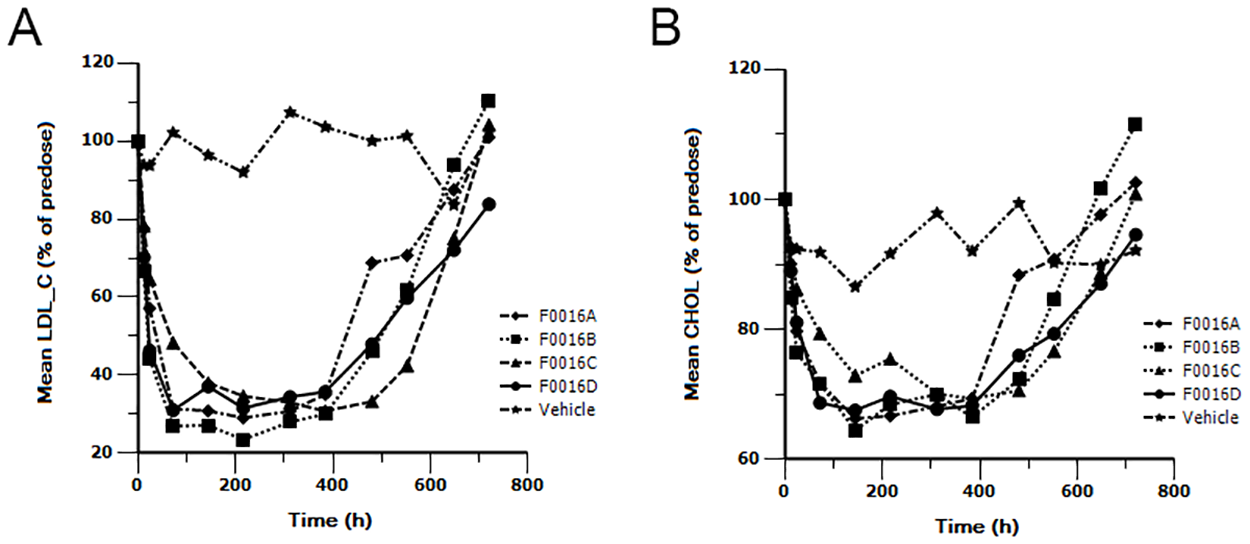
LDL-C (A) and CHOL (B) analyses after a single intravenous dose of 5 mg/kg F0016 mAbs to cynomolgus monkeys. Points, mean of two animals. Serum samples were analyzed at different points in time (day 1 ~ day 31) for lipid measurements using the automatic biochemical analyzer.

**Fig 4:**
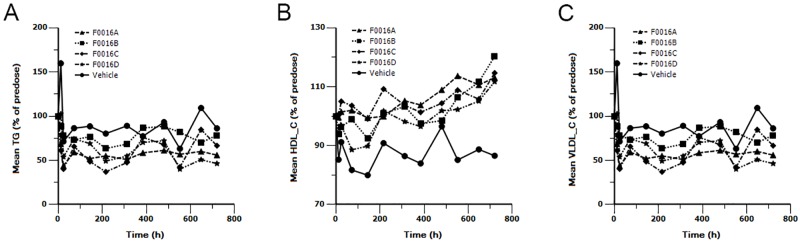
TG (A), HDL-C (B) and VLDL-C (C) analyses after a single intravenous dose of 5 mg/kg F0016 mAbs to cynomolgus monkeys. Points, mean of two animals. Serum samples were analyzed at different points in time (day 1 ~ day 31) for lipid measurements using the automatic biochemical analyzer.

To explore whether the improved PK profiles are associated with improved duration of efficacy, lipid levels were measured in cynomolgus monkeys. After a single intravenous injection of 5 mg/kg F0016A, F0016B, F0016C and F0016D, plasma levels of LDL-C (Fig 3A) and CHOL (Fig 3B) were both reduced, While no obvious trend changes in TG (Fig 4A), HDL-C (Fig 4B) and VLDL-C (Fig 4C) were found after dosing. Compared to the respective baseline of LDL-C before administration, the maximal lipid-lowing effect (E_max_) of the four mAbs were similar (Table 5). However, as expected, there was a visible difference in the time to maintain the effect when 40% of LDL-C inhibition was defined as a significant lipid-lowing effect. In detail, the efficacy of F0016A, F0016B, F0016C and F0016D for profoundly inhibiting the LDL-C level (about 40%) began to disappear at 480 h, 552 h, 648 h and 648 h after administration, respectively (Table 6). Again, the lipid-lowing effect of F0016C and F0016D maintained longer than F0016A (168h) and F0016B (96h), consistent with a trend of increased half-lives in PK study.

**Table 5:** PD parameters of WT anti-PCSK9 antibody (F0016A) and the Fc variants (F0016B, C, D) after a single intravenous dose of 5 mg/kg to cynomolgus monkeys. PD parameters (E_max_ and T_max_) was obtained by visible inspection of PD data.

Antibody		CHOL mmol/L	TG mmol/L	LDL-c mmol/L	HDL-c mmol/L	VLDL-c mmol/L
F0016A	E_max_	2.06	na	0.40	na	na
	T_max_	144h(D7)	na	216h(D10)	na	na
F0016B	E_max_	2.17	na	0.37	na	na
	T_max_	144h(D7)	na	216h(D10)	na	na
F0016C	E_max_	2.61	na	0.49	na	na
	T_max_	384h(D17)	na	384h(D17)	na	na
F0016D	E_max_	2.27	na	0.48	na	na
	T_max_	144h(D7)	na	72h(D4)	na	na

na, not applicable.

**Table 6:** Changes in serum LDL-C (% of predose) after a single i.v. administration of 5 mg/kg WT anti-PCSK9 antibody (F0016A) and the Fc variants (F0016B, C, D) to cynomolgus monkeys.

Time points (h)	Vehicle (%)	F0016A (%)	F0016B (%)	F0016C (%)	F0016D (%)
Predose	na	na	na	na	na
12	93.09	76.71	67.10	78.48	69.87
24	92.55	56.16	44.52	64.56	46.15
72	101.60	30.82	27.10	48.73	30.77
144	95.74	29.45	27.10	37.97	37.18
216	91.49	27.40	23.87	34.81	31.41
312	106.38	29.45	28.39	33.54	34.62
384	103.19	34.93	30.32	31.01	35.90
480	99.47	71.23	45.81	33.54	48.72
552	101.06	73.29	61.94	42.41	60.90
648	83.51	89.04	93.55	74.05	73.08
720	102.13	100.68	110.32	102.53	84.62

na, not applicable.

## Discussion

PCSK9 plays an important role in lipid metabolism. Blocking PCSK9 binding to LDLR can profoundly lower plasma LDL levels. Two PCSK9 mAbs, alirocumab and evolocumab, are currently available for treatment in adult patients with heterozygous familial or patients with clinical atherosclerotic cardiovascular disease. Alirocumab is given every two weeks by subcutaneous injection at a dose of 75–150 mg, and the recommended dose of evolocumab is 140 mg every two weeks or 420 mg once monthly [26,27]. Both drugs are fully human mAbs and thus have a minimal risk of triggering an immune response to the antibody [12]. Several studies have demonstrated similar trends in safety and efficacy profiles, including significant reductions in LDL-C and lipoprotein [28,29].

Beyond fundamental demonstrations of safety and efficacy, additional considerations in the development of mAbs against PCSK9 have been the search for prolonged dosing intervals, longer half-lives, and greater ease of administration. In addition to increasing the affinity for FcRn by Fc mutation, which is the most widely studied approaches, the main methods used to increase the half-lives of mAbs are: modification by PEG [30]; reduction of CL of the immune complex by pH-sensitive binding to the antigen [31]; reduction of Ab CL by decreasing the PI of the variable region [32].

In this study, we evaluated the antigen-binding activity and binding to FcRn in vitro of F0016A (alirocumab) and three Fc mutants: F0016B, F0016C and F0016D. The results of the antigen-binding assay showed that the Fc mutation had no effect on Fab function. By contrast, the mAbs exhibited approximately 2-fold lower affinity to the D374Y variant (a gain-of-function mutation) than to WT PCSK9. Gain-of-function mutations in PCSK9 result in increased PCSK9 function, which leads to decreased LDLR recycling to the cell surface. This results in autosomal-dominant hypercholesterolemia with increased plasma LDL levels and increased risk of developing coronary heart disease [33, 34]. Therefore, improve the potency or increasing the half-life of therapeutic mAbs may benefit patients bearing PCSK9 gain-of-function mutations. Next, the FcRn binding assay confirmed that the three Fc mutants of alirocumab had enhanced affinity to FcRn, consistent with previous reports. F0016C had the strongest affinity to FcRn, suggesting that it might have the longest half-life *in vivo*.

*In vivo* PK and PD evaluations of the four F0016 mAbs were performed in cynomolgus monkeys. The half-lives of the different F0016 mAb candidates were calculated by non-compartmental method towards the linear section of time-concentration curve. The nonlinear section of terminal elimination may be explained by interaction with target antigen. However, the DNA sequence for mAb Fab section and cell line used for production of 4 candidates—anti-PCSK9 WT and the three Fc variants were identical, and the antigen-binding measurement *in vitro* for wild and modified mAbs showed similar results. Therefore, target-mediated nonlinear elimination effect was considered to be consistent for the 4 candidates, increased half-lives were more likely relevant to enhanced effect of Fc-modified human PCSK9 monoclonal antibodies. The PK and PD evaluation showed that F0016C and F0016D exhibited longer half-lives and maintained much longer lipid-lowing effect time than the other two mAbs. In addition, the individual differences in the lipid-lowering effect at each time point were smaller in the groups of animals that received F0016B and F0016C, and there were large deviations at some time points in F0016A and F0016D animals. Corresponding differences in the lipid-lowering effects and blood concentrations in the corresponding animals were observed at each time point. However, individual differences could not be assessed because each group had only 2 animals.

The *in vitro* and *in vivo* studies of the four PCSK9 mAbs confirmed that the half-lives of the three Fc mutants of alirocumab were prolonged to different degrees, and the lipid-lowering effect was also improved. The corresponding molecular mechanism involves the increased affinity of the Fc segment to FcRn and increased pH-dependent binding to FcRn. Previous research attempts to increase antibody half-life by mutating the Fc region at important sites required for FcRn binding have yielded relative success [35]. It is reasonable to expect that mAbs with longer serum half-lives may also be engineered by transferring the Fc mutations into different IgG subtypes. In addition, it should now also be possible to alter the serum half-lives of other IgG-related therapeutics such as IgG Fc fusion proteins using this approach.

Engineered mAbs with increased serum half-lives might prove valuable in antibody therapy. For example, it may be possible to reduce the frequency of administration of such mAbs. This will be a great benefit to patients undergoing long-term antibody therapy. Prolonged half-lives of therapeutic antibodies have been reported to ultimately reduce administration frequency or dosage requirements while maintaining or improving the efficacy of an antibody [36]. Based on the results described in this study, F0016C, which is considered an ideal long-acting PCSK9 mAb candidate, may be effective in the future at lowering LDL-C with a lower frequency of administration or drug dose, thereby reducing the potential side effects of the drug and the burden on the patient. Thus, Fc-modified mAbs with longer serum half-lives should represent a potent new class of human therapeutics.
